# Are there sustained psychological impacts in women diagnosed with in-situ or early invasive breast cancers?

**DOI:** 10.3389/fgwh.2022.763174

**Published:** 2023-01-16

**Authors:** Bettina Braun, Joke Tio, Barbara Krause-Bergmann, Hans-Werner Hense

**Affiliations:** ^1^ Institute of Epidemiology and Social Medicine, University of Münster, Münster, Germany; ^2^ Institute for Cancer Epidemiology, University of Lübeck, Lübeck, Germany; ^3^ Breast Care Center, Department of Gynecology and Obstetrics, University Hospital Münster, Münster, Germany; ^4^ Department for Breast Diseases, St Franziskus Hospital, Münster, Germany

**Keywords:** screening mammography, overdiagnosis, quality of life, depression and anxiety, breast cancer

## Abstract

**Purpose:**

The detection of a ductal carcinoma in-situ (DCIS) or an early invasive breast cancer (EIBC), particularly by population-wide mammography-screening-programs, is controversial as an unknown proportion of these cases may be due to overdiagnosis. We investigated whether women with such potentially overdiagnosed breast cancers suffer from sustained adverse psycho-social consequences.

**Methods:**

Standardized questionnaires were mailed to 900 survivors, diagnosed with either DCIS or EIBC, requesting self-reports on quality of life using EORTC Quality of Life Questionnaire C-30. Levels of anxiety and depression were assessed using the HADS questionnaires. Item score values in the study group were compared to reference data obtained from normative studies in the German female reference population.

**Results:**

The 577 women who returned completed questionnaires had a mean age of 65.1 years, 387 (67%) had been diagnosed by mammography screening. Median time since diagnosis was 5.9 years. There were no substantial differences between the study sample and the reference population for most of the items. While most score values were even slightly more favorable in the study group, the scores for cognitive function were moderately lower, especially among younger patients. Score values for anxiety were generally higher among younger women (50 to 59 years) from the study group, while depression scores were lower irrespective of age.

**Conclusions:**

This study indicates that the diagnosis of DCIS or EIBC, which is predominantly a result of screening, does not seem to induce sustained, adverse psychological impacts in affected women when compared with the respective general female population. Only anxiety levels remained elevated among younger women.

## Introduction

The numbers of women who survive a diagnosis of breast cancers have risen consistently over recent years (1) and correspondingly the interest in breast cancer survivorship and, particularly, in the quality of life has increased (2, 3). Results are inconsistent and some studies show that the overall quality of life of breast cancer patients decreases immediately after diagnosis but improves over time and with the termination of therapies (4). Presently, breast cancer screening by population-wide mammography screening programs is propagated in many countries: these programs aim to advance the diagnosis of a breast cancer diagnosis by regular invitations to standardized mammography examinations. Because of screening, many women obtain diagnoses of breast cancers that are either in-situ carcinomas (DCIS) or early invasive breast cancer (EIBC), which are less than 20 mm in diameter with no lymph node or distant tissue involvement (i.e., T1N0M0 in the international TNM classification system). The European Guidelines for Quality Assurance in Breast Cancer Screening and Diagnosis (5) emphasize that, apart from saving lives, early tumor detection leads also to less invasive and stressful cancer therapies and that this should result in a sustained beneficial psycho-social impact of screening. However, because of the high detection rates of these very early cancer stages, mammography screening programs tend to create substantial numbers of potential overdiagnoses, that is, the identification of slow-growing indolent breast tumors which would probably have remained undetected without mammography screening (6–8). It has been suggested that, considering the large numbers of women who are potentially overdiagnosed with breast cancer as a result of screening, the overall impact on quality of is likely substantial (9, 10).

To date, only few studies have attempted to quantify such an impact from empirical data. Single studies investigated the quality of life of women with in-situ and early invasive cancers and compared them to women without breast cancer: they observed an initially lower quality of life which improved with increasing time since diagnosis (11). The authors of a systematic review comprising numerous and but short studies of women with in-situ carcinomas identified the need for more well-powered studies that should focus on the long-term psycho-social impact in these women (12).

The present report investigated whether there are sustained psycho-social consequences for women diagnosed with in-situ or early invasive breast cancer by comparing indicators for quality of life and psycho-social function as well as symptoms of depression or anxiety to age-stratified normative reference samples from the German female population (13, 14).

## Materials and methods

The initial sample for this retrospective analysis consisted of 936 women, aged between 50 and 69 years, who had been diagnosed with either an incident ductal in-situ carcinoma (*N* = 286 [30.6%]) or with an incident early invasive breast cancer (T1N0M0; *N* = 650 [69.4%]) in two breast care centers in the city of Münster, Germany, between 2006 and 2012 (15). Information on the date of diagnosis and the primary therapeutic management was extracted from the certified clinical tumor documentation system operative in both breast care centers. The mode of cancer detection (by mammography screening or clinical diagnosis) and the present vital status for each individual cancer case was identified by linkage with the population-based State Cancer Registry of North Rhine-Westphalia (LKR NRW) (15).

Questionnaires were mailed to 900 women still alive in 2015. Data on sociodemographic variables, current comorbidities such as depression or diabetes, type of first-line breast cancer therapy, recent course of the disease, and current breast cancer therapy were obtained from self-reports and refer to the time of filling in the mailed questionnaires.

The standardized questionnaire of the European Organization for Research and Treatment of Cancer (EORTC C30, Version 3.0) were used to obtain score values for overall quality of life (QoL), cognitive function (CF), emotional function (EF), physical function (PF), role function (RF), and social function (SF) (16, 17). As the questionnaires were mailed an average of five and more years after the cancer diagnosis, questions about current symptoms were also omitted assuming that they were primarily relevant in the early phase of the primary treatment. The functional scales were scored with values from 0 to 100 with higher scores corresponding to better functioning. Th score values were analyzed based on the scoring manual (17). Score differences of more than 10 points were regarded as substantial (16, 18, 19). We compared the study results with the age-stratified mean score values from a normative reference study (*N* = 4,684) representative for the German general population which had been provided for use as a comparison of health-related quality of life data in German cancer patients (14).

Information on the degree of depression and anxiety among women of the study sample were obtained with the Hospital Anxiety and Depression Scale (HADS), a 14-items checklist in which seven items relate to depression and seven to anxiety (20). The item values range from 0 to 3 and anxiety and depression scores are obtained by summing up the scores over the seven items, yielding values between 0 and 21. Scores values of 8 or above were considered as a raised level of anxiety or depression. Normative values from a representative sample of the German general population (*N* = 4,410) were available for an age-stratified comparison with the present study (13).

## Statistical methods

Baseline characteristics describe the study sample based on self-reported socio-demographic variables, breast cancer treatment up to the time of interview and current comorbidity. Mean values, and their standard deviations, calculated for overall quality of life, breast and body image, and for the five functional scores in 10-years age groups were compared to the normative values of the respective age groups of women in the German general population. Differences of mean score values are reported with their 95% confidence intervals. Similarly, the age-stratified mean values for the scores of anxiety and depression were compared to mean reference values of the respective normative sample of the German general population, and the differences in mean values are also reported with their 95% confidence intervals. Because this analysis is strictly explanatory and comparative, no statistical significance values are reported. The 95% confidence intervals were only used a measure of precision of the reported estimates (15). The statistical software programs SAS 9.4 and MedCalc V19.6 were used for the analyses.

## Results

Completed questionnaires were returned by 577 women (response 64.1%) who had either a diagnosis of a ductal carcinoma in-situ (*N* = 122) or an early invasive breast cancer (*N* = 455). Their average age when returning the questionnaire was 65.1 years, and approximately one quarter of the women was younger than 60 years. Two of three women (*N* = 387) had been detected by mammography screening and one third was diagnosed clinically. The median time span from diagnosis to filling in the questionnaire was 5.9 years. About three out of four women had been treated with breast conserving surgery and/or with radiation (Table 1).

**Table 1:** Baseline characteristics of 577 women with ductal carcinoma in-situ or early invasive breast cancer, who returned the questionnaires.

		*N* (%)
Age at interview	50–59 years	122 (21.1%)
	60–69 years	455 (78.9%)
	Mean age (SD)	65.1 (6.3)
Participation in mammography screening program	Yes	387 (67.1%)
	No	190 (32.9%)
Time since diagnosis	<5 years	202 (35.0%)
	≥5 years	375 (65.0%)
	Mean time (SD)	5.9 (2.0)
Education	<11 years	226 (39.2%)
	≥11 years	343 (59.4%)
	Missing	8 (1.4%)
Marital status	Living alone	154 (26.7%)
	Living with partner	418 (72.4%)
	Missing	5 (0.9%)
Self-reported comorbidities		
Diabetes mellitus	Yes	39 (6.8%)
	No	529 (91.7%)
	Missing	9 (1.6%)
Depression (ever)	Yes	98 (17.0%)
	No	468 (81.1%)
	Missing	11 (1.9%)
Chronic obstructive lung disease	Yes	52 (9.0%)
	No	517 (89.6%)
	Missing	8 (1.4%)
Osteoporosis	Yes	84 (14.6%)
	No	477 (82.6%)
	Missing	16 (2.8%)
Breast cancer treatment		
Surgical procedure	Breast conserving	430 (74.5%)
	Mastectomy	146 (25.3%)
	Missing	1 (0.2%)
Radiation	Yes	412 (71.4%)
	No	158 (27.4%)
	Missing	7 (1.2%)
Hormone therapy	Yes	209 (36.2%)
	No	361 (62.6%)
	Missing	7 (1.2%)
Under breast cancer treatment at time of interview	Yes	171 (29.6%)
	No	400 (69.3%)
	Missing	6 (1.04%)

Comparing the mean score values for overall quality of life and cognitive, emotional, physical, social and role function with the respective mean values from the German general reference population, there were no substantial (more than 10 score points) differences between the study sample and the reference population for any of the items (Table 2). Interestingly, most score values were generally even slightly more favorable for women from the study sample than for those in the reference population. Only the scores for cognitive function were moderately lower among women with in-situ and early invasive breast cancers and this was slightly more pronounced in those aged 50 to 59 years.

**Table 2:** Mean values (and standard deviation, SD) of scores values among women with ductal carcinoma in-situ or early invasive breast cancer and the reference study.

	Women with DCIS or EIBC	Reference Study*	Mean Difference and 95% Confidence Interval
50–59 years	*N* = 122	*N* = 414	
Mean QoL (SD)	69.5 (22.3)	63.2 (25.4)	+6.3 [+11.3; +1.3]
Mean CF (SD)	73.5 (25.8)	81.3 (24.5)	−7.8 [−12.8; −2.8]
Mean EF (SD)	60.6 (28.4)	62.2 (28.0)	−1.6 [−7.3; +4.1]
Mean PF (SD)	85.8 (15.9)	82.6 (21.5)	+3.2 [−0.9; +7.4]
Mean RF (SD)	78.8 (27.9)	75.0 (31.9)	+3.8 [−2.5; +10.1]
Mean SF (SD)	75.4 (30.0)	78.4 (29.6)	−3.0 [−9.0; + 3.0]
60–69 years	*N* = 455	*N* = 412	
Mean QoL (SD)	70.7 (20.1)	65.5 (24.5)	+5.2 [+2.2; +8.2]
Mean CF (SD)	85.7 (20.1)	87.1 (19.3)	−1.4 [−4.0; +1.2]
Mean EF (SD)	73.9 (23.6)	72.8 (24.9)	+1.1 [−2.1; +4.3]
Mean PF (SD)	84.0 (17.3)	81.0 (21.0)	+3.0 [+0.4; +5.6]
Mean RF (SD)	82.7 (24.0)	77.3 (29.3)	+5.4 [+1.8; +9.0]
Mean SF (SD)	86.2 (22.3)	83.1 (25.8)	+3.1 [−0.1; +6.3]

DCIS, ductal carcinoma in-situ; EIBC, early invasive breast cancer; QoL, quality of life; CF, cognitive functioning; EF, emotional functioning; PF, physical functioning; RF, role functioning; SF: social functioning.

* Waldmann et al. (14).

As compared to the female German general population of the same age, the mean values for anxiety were generally slightly higher in women with in-situ and early invasive breast cancers (Table 3). Likewise, the proportion of women with score values above the threshold of 8 points (i.e., a raised level of anxiety) was 30% in women with in-situ and early invasive breast cancers as compared to 25% in the general population. By contrast, the mean score values for depression were consistently lower among women in the study sample such that the proportion of women with a raised degree of depression (>8 score points) was only about 10% which was clearly lower than the approximately 30% reported for the general population.

**Table 3:** Mean values (and standard deviation, SD) of hospital anxiety and depression scale (HADS) scores among women with DCIS or EIBC and the reference study.

	Women with DCIS or EIBC	Reference Study*	Mean Difference and 95% Confidence Interval
50–59 years	*N* = 122	*N* = 410	
Mean Anxiety Score (SD)	7.2 (4.5)	5.4 (3.6)	+1.8 [+1.0; + 2.6]
Mean Depression Score (SD)	4.4 (4.3)	5.0 (3.7)	−0.6 [−1.4; + 0.2]
60–69 years	*N* = 455	*N* = 417	
Mean Anxiety Score (SD)	5.4 (3.9)	5.1 (3.6)	+0.3 [−0.2; + 0.8]
Mean Depression Score (SD)	3.3 (3.4)	5.4 (3.8)	-2.1 [−2.6; −1.6]

DCIS, ductal carcinoma in-situ; EIBC, early invasive breast cancer.

* Hinz and Brähler (13).

## Discussion

The present study compared a study group of 577 women with in-situ and early invasive breast cancer with independent samples from normative reference studies of the respective German general population: five to six years after the cancer diagnoses, the differences in scores of quality of life, functional parameters, and anxiety and depression were generally only moderate or absent. Presuming that a large proportion of women with in-situ and early invasive breast cancer is potentially overdiagnosed, the present study seems to suggest that such potential overdiagnoses had only minor sustained, adverse psychological consequences.

Of note, however, more than five years after the diagnosis, the scores for cognitive function were consistently lower in women with early breast cancers, in particular when they were younger, that is, aged 50 to 59 years. Cognitive constraints have been observed in various studies as sustained effects of anticancer treatment (21). There are reports hypothesizing that adverse cognitive effects may be aggravated by individual factors such as stress (22). Interestingly, the younger women in our study produced a second noticeable result: their level of anxiety was generally moderately raised. This has been associated before in other studies with fear of tumor recurrence (4, 12, 23, 24). Thus, it appears conceivable that diagnosis and treatment of in-situ and early invasive breast cancer may cause slight sustained adverse responses. However, the magnitude of these differences did not surpass predefined thresholds of clinical relevance.

The score values for depression and the prevalence of raised levels of depression were much lower in the study group than in the general population. Supposedly, this finding is due to a characteristic of the study group already observable at the outset (Table 1) as the self-reported life-time depression prevalence was much lower (17.3%) than that (30%) in the reference study (13).

About two thirds of women in the study sample had been detected in the population-wide mammography screening program. However, since in-situ and small invasive breast cancers below 20 mm diameter are commonly impalpable and mostly symptomless, we suggest that the study participants detected outside of the screening program were presumably attributable to masked or opportunistic (“grey”) screening activities. We suppose, therefore, that the women in this study sample were almost entirely diagnosed by some sort of screening.

Several study limitations need to be addressed. This report is based on a series of all breast cancer patients treated in two certified breast cancers in one German city between 2006 and 2012; therefore, the limited generalizability of the results to other populations needs to be considered. Furthermore, the potential impact of novel treatment options introduced since 2016 and their impact on sustained psycho-social sequelae will not be reflected by the study results. The response rate to the mailed questionnaires was rather low which may have introduced a certain level of self-selection into the study sample. Moreover, low numbers for women with in-situ and early invasive breast cancer in the age group 50 to 59 years compromise the precision of the estimates provided for the score value differences, i.e., the width of the 95% confidence intervals, in this age group Another limitation should be considered when regarding the results of the EORTC: although we only used the relevant age groups when comparing our results with the reference group, the age structure within the subgroups still could be different. In addition, potential confounding factors such as comorbidities or socioeconomic components could not be taken into account as these are not equally available in the reference and our study.

In conclusion, women with in-situ and early invasive breast cancer, which is commonly detected by mammography screening, do Klicken oder tippen Sie hier, um Text einzugeben.not seem to be affected by sustained psychological harm five or more years after diagnosis. Specifically, concerns considering the long-term psycho-social effects of related potential overdiagnoses were not confirmed.

## Data Availability

The raw data supporting the conclusions of this article will be made available by the authors, without undue reservation.
